# Experimental Investigation on Flexural Behavior of Reinforced Concrete Beams with Externally Applied Liquid Rubber

**DOI:** 10.3390/ma19173796

**Published:** 2026-09-06

**Authors:** Qi Ouyang, Xian Liang, Lvkang Lan, Weizhu Zhu, Xianxiang Zhou

**Affiliations:** College of Civil Engineering and Architecture, Quzhou University, Quzhou 324000, China; liangxian@qzc.edu.cn (X.L.); 38109@qzc.edu.cn (L.L.); w.z.zhu@outlook.com (W.Z.); zhouxianxiang1@163.com (X.Z.)

**Keywords:** reinforced concrete beam, liquid rubber, crack resistance, ductility, flexural behavior

## Abstract

This paper presents a technique for enhancing the cracking resistance of reinforced concrete (RC) beams through the external application of liquid rubber. To evaluate the influence of this coating on flexural performance, four-point bending tests were conducted on seven RC beams, comprising six coated beams and one uncoated control beam. The effects of coating position (beam soffit, beam sides, or both) and number of coating layers (3 or 6) on the flexural response were investigated. The results indicate that the external application of liquid rubber improves the cracking load and ductility of the RC beams to some extent, and the degree of improvement is related to the number of coating layers. Moreover, the application of liquid rubber to the tensile zone of the RC beams can moderately restrain tensile deformation of the concrete. When the coating was applied only to the beam soffit, the concrete strain at the soffit was lower than that in the tensile zone on the beam sides, resulting in a non-linear distribution of tensile strain along the section height within the pure bending region.

## 1. Introduction

Reinforced concrete (RC) structures are widely used in various engineering applications due to their low construction cost and good durability [1,2]. However, with the extension of service time and long-term exposure to aggressive environments and complex loading conditions, RC structures are prone to cracking. The initiation and propagation of cracks not only reduce the stiffness of the members but also provide pathways for the ingress of harmful agents, accelerating steel corrosion and concrete deterioration, which seriously compromise the durability and serviceability of the structures [3,4,5,6].

To improve the crack resistance of RC structures, several technical strategies have been proposed, including fiber-reinforced concrete (FRC), externally bonded fiber-reinforced polymer (FRP) composites, and textile-reinforced concrete (TRC). FRC incorporates steel [7,8,9], polypropylene [10,11,12], or basalt fibers [13,14] into the cementitious matrix, leveraging the fiber bridging effect to restrain crack initiation and propagation. Externally bonded FRP materials, characterized by excellent mechanical properties, are widely adopted for strengthening RC members, effectively enhancing load-carrying capacity and restricting crack development [15,16,17]. TRC, which combines textile reinforcements with fine-grained concrete, exhibits favorable crack resistance, thermal stability, and compatibility with concrete substrates [18,19,20]. Nevertheless, these approaches have inherent limitations: the performance of FRC is highly dependent on construction quality and fiber dispersion; FRP strengthening relies on epoxy adhesives, which are prone to interfacial debonding under hygrothermal cyclic conditions [21,22]; and TRC is often subject to debonding between the textile and the concrete matrix, limiting full utilization of the textile tensile strength [23,24]. Hence, there remains a pressing need for the development of novel and efficient anti-cracking techniques for RC structures.

Liquid rubber is a polymeric material characterized by excellent adhesion, crack resistance, and tensile deformability. In recent years, it has found preliminary applications in structural strengthening and crack mitigation. Several studies have incorporated liquid rubber as a modifier into epoxy adhesives for bonding FRP sheets to reinforce RC members. For instance, Gao et al. [25] found that liquid rubber-modified epoxy adhesive used for bonding CFRP sheets significantly improved both the ultimate load capacity and ductility of RC beams. Pang et al. [26,27] investigated the effects of liquid rubber-modified adhesive on the bond performance and durability of CFRP–steel interfaces, demonstrating enhanced interfacial bond strength and improved durability. However, in these studies, liquid rubber served only as an auxiliary additive to the adhesive, and its contribution to the crack resistance of RC members depended on the synergistic action with FRP and the adhesive. To date, limited research has addressed the direct application of liquid rubber as an independent spray-coated protective layer on RC beam surfaces for crack resistance enhancement. In contrast to its role as an adhesive modifier, a directly sprayed liquid rubber coating can fully exploit its high elasticity and large deformation capacity, accommodating crack opening through compatible deformation, thus constituting an effective protective barrier on the beam surface. Moreover, liquid rubber can be applied via spraying, offering advantages such as rapid curing, ease of construction, and dry-operation processing, which render it attractive for practical engineering applications.

Against this background, the present study proposes the direct spraying of liquid rubber onto RC beam surfaces as an anti-cracking coating. Four-point bending tests were conducted to examine the effects of different coating locations and layer numbers on the flexural behavior of RC beams. The influence of externally applied liquid rubber on crack resistance, load-carrying capacity, and deformability was systematically analyzed, with the aim of providing a novel technical pathway for crack protection of RC structures.

## 2. Experimental Program

### 2.1. Specimen Design

**Figure 1:**
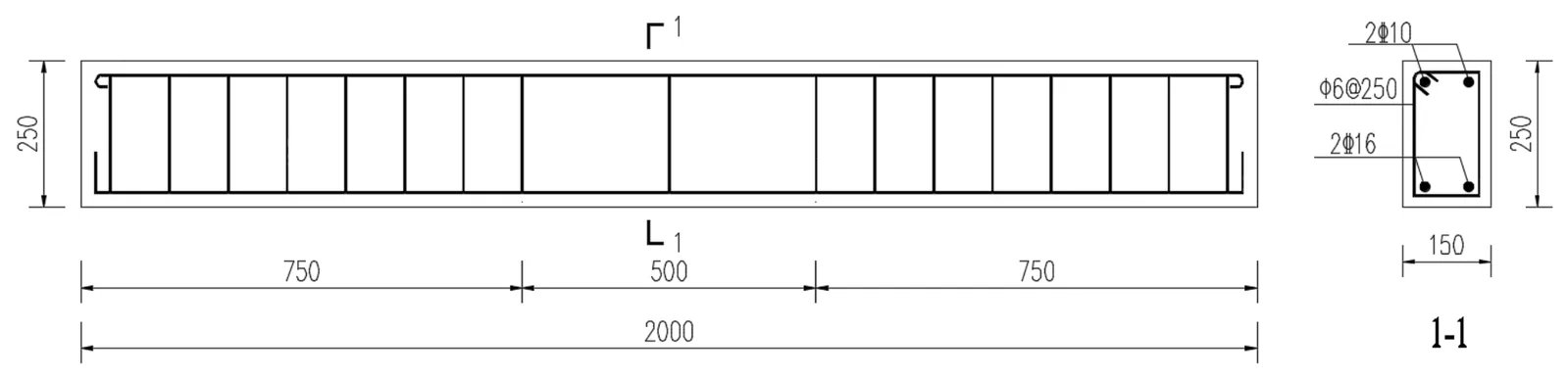
Dimensions and reinforcement details of the test beams (unit: mm).

A total of seven reinforced concrete beams were designed in this experimental study, all sharing the same dimensions and reinforcement configurations. Each beam had a length of 2000 mm, a width of 150 mm, and a height of 250 mm. The design strength grade of the concrete was C25, and the clear cover for the reinforcement in the beam was 25 mm. HRB400-grade steel bars were used as the longitudinal reinforcement, and HPB300-grade steel bars were employed as stirrups. The bottom longitudinal reinforcement comprised two bars with a diameter of 16 mm, and the top longitudinal reinforcement consisted of two bars with a diameter of 10 mm. The stirrups, which had a diameter of 6 mm, were arranged with a spacing of 100 mm in the shear-bending zones and 250 mm in the pure bending zone. The dimensions and reinforcement arrangement of the beams are shown in Figure 1.

The liquid rubber coating configurations applied to the reinforced concrete beams are summarized in Table 1. Beam L1 was designated as the control specimen and received no liquid rubber coating. Beams L2 and L3 were coated with three and six layers of liquid rubber, respectively, on their bottom surfaces. Beams L4 and L5 received three and six layers, respectively, on the lower half of their side surfaces. Beams L6 and L7 were coated with three and six layers, respectively, on both the bottom surfaces and the lower half of the side surfaces. The liquid rubber was applied at a thickness of 0.2 mm per layer, and the coating rate was maintained at 100% for each layer. Following the coating application, all beam surfaces appeared black. To enable clear observation of cracks during testing, a white latex paint was brushed onto the beam surfaces, and black grid lines with a spacing of 50 mm × 50 mm were marked on them.

### 2.2. Materials

#### 2.2.1. Concrete

In this study, concrete with a strength grade of C25 was employed, consisting of cement, fine aggregate, coarse aggregate, and water, with a water-to-cement ratio (w/c) of 0.5. Jiangshanhu brand P·P32.5 Portland-pozzolana cement (Jiangshan Hu Group Co., Ltd., Jiangshan, Zhejiang, China) was adopted as the cementitious material. The fine aggregate was natural river sand, which was classified as medium sand with a favorable gradation. The coarse aggregate was crushed gravel with a maximum nominal size of 20 mm. Ordinary tap water was used for mixing. All test beams were cast in a single batch to ensure material consistency. During beam casting, concrete specimens were prepared as supplementary samples and cured under the same conditions as the test beams, in order to measure the cubic compressive strength and elastic modulus of the concrete. In accordance with GB/T 50081-2019 [28], the cubic compressive strength f_cu_ was determined to be 26.9 MPa, and the elastic modulus E_c_ was 29.3 GPa. The detailed mix proportion and the resulting mechanical properties are summarized in Table 2.

#### 2.2.2. Liquid Rubber

The liquid rubber employed in this study is a multifunctional synthetic polymer liquid rubber, supplied by Bolier (Beijing) Technology Development Co., Ltd. (Beijing, China). This material is a waterborne two-component system that combines rapid curing characteristics and is suitable for use as a hyperelastic waterproof and anti-cracking coating. In this system, Component A consists of ultrafine suspended micro-emulsified modified asphalt and synthetic polymer, while Component B is a special calcium chloride-based curing agent. Upon atomized spraying, the two components come into contact with each other on the substrate surface and rapidly cure to form a solid film. As the curing reaction proceeds and moisture continuously evaporates, the coating film gradually evolves into a durable waterproof and anti-cracking protective layer characterized by high elasticity, enhanced toughness, and excellent aging resistance. In accordance with GB/T 16777-2008 [29], the measured bonding strength of the liquid rubber was 0.9 MPa, the tensile strength was 1.90 MPa, and the elongation at break was 870%. The liquid rubber exhibited a curing time of 1.63 h, a heat resistance of 110 °C, and a density of 0.981 g·cm^−3^. The detailed physical and mechanical parameters are summarized in Table 3.

#### 2.2.3. Reinforcement

**Figure 2:**
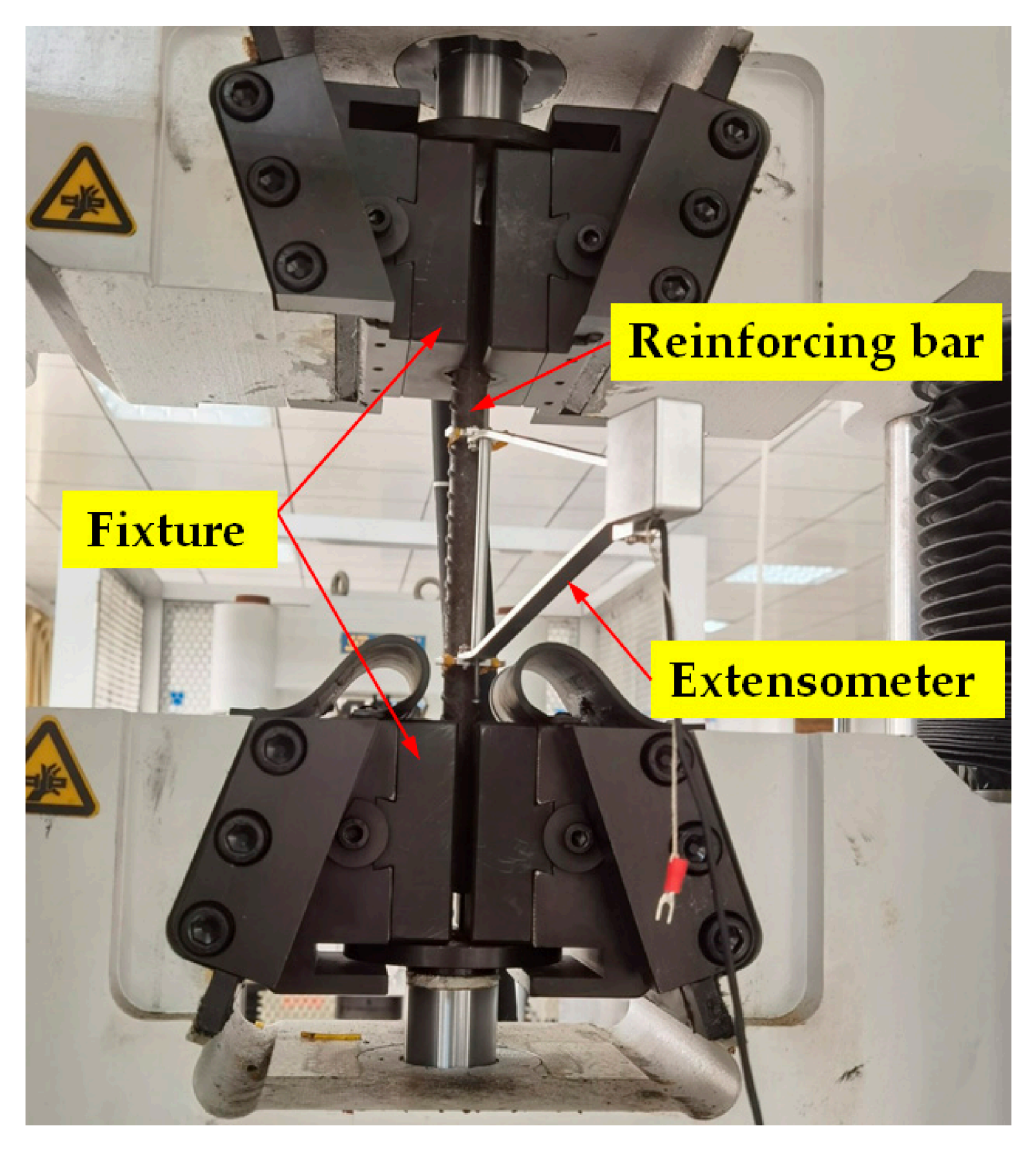
Tensile test of reinforcing bars.

Tensile tests were conducted on the longitudinal reinforcement and stirrups using a universal testing machine (Shanghai Hualong Testing Instruments Co., Ltd., Shanghai, China) in accordance with GB/T 228.1-2021 [30], as shown in Figure 2. The yield strength, tensile strength, elastic modulus, and elongation after fracture were determined through the tests, and the complete mechanical properties of the reinforcement are summarized in Table 4.

### 2.3. Specimen Preparation

**Figure 3:**
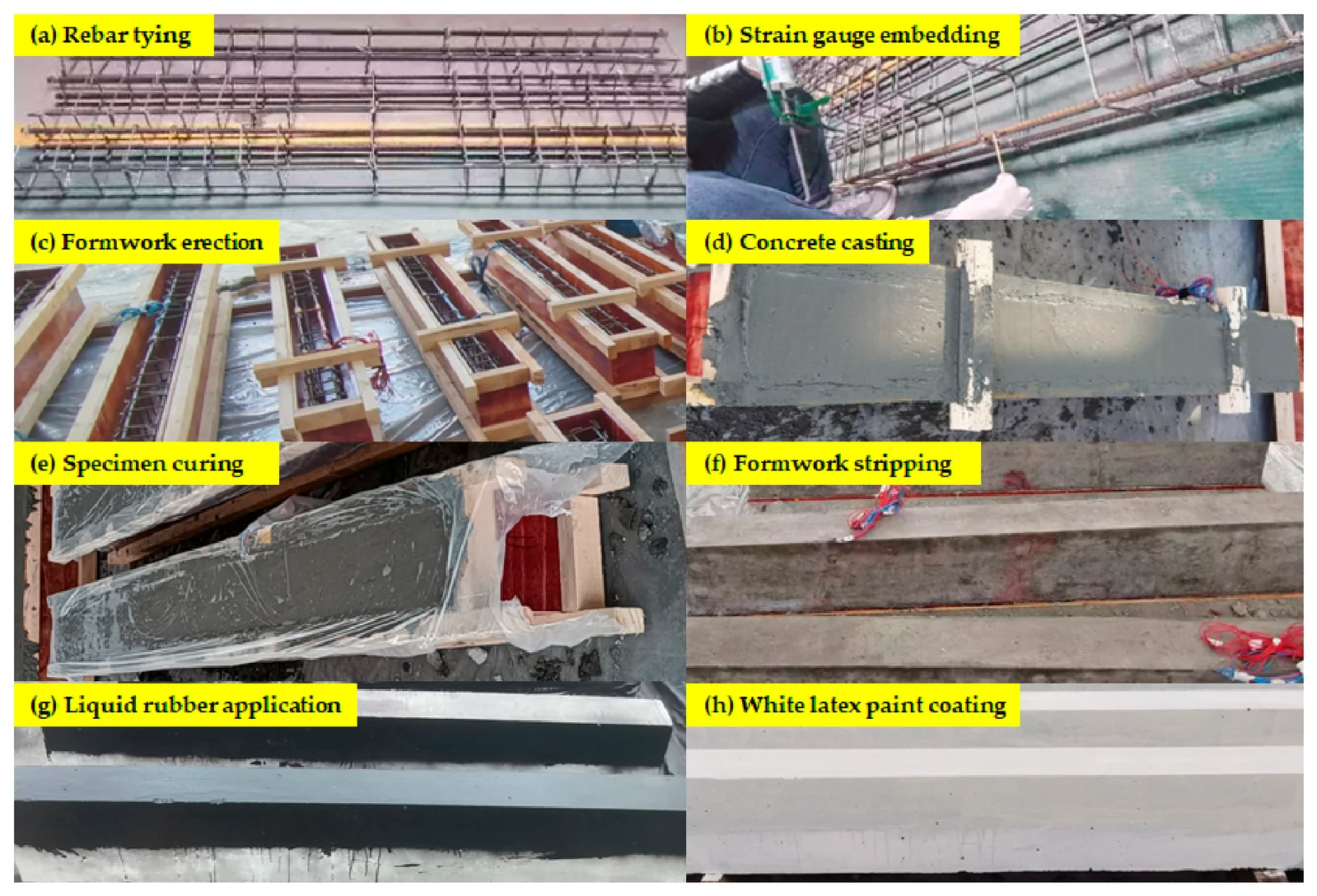
Preparation of the reinforced concrete test beams externally coated with liquid rubber: (**a**) Rebar tying; (**b**) Strain gauge embedding; (**c**) Formwork erection; (**d**) Concrete casting; (**e**) Specimen curing; (**f**) Formwork stripping; (**g**) Liquid rubber application; (**h**) White latex paint coating.

The preparation procedure for the reinforced concrete test beams externally coated with liquid rubber is presented in Figure 3. The specimens were fabricated through a series of sequential operations, including rebar tying, strain gauge embedding, formwork erection, concrete casting, specimen curing, formwork stripping, liquid rubber application, and final coating with white latex paint. After 28 days of concrete curing, the surfaces of the test beams to be coated with liquid rubber were subjected to cleaning treatment, and the liquid rubber was subsequently applied using a dedicated spraying apparatus. Prior to spraying, the test beams were arranged in a row and coated in a single batch to ensure uniform coating thickness across all specimens. The liquid rubber was applied using a multi-layer spraying process, with each layer allowed to air-dry for 24 h before the subsequent layer was applied. To guarantee thickness uniformity between layers, the required amount of liquid rubber for each layer was pre-calculated, and both the dosage and spray gun flow rate were kept constant across all layers. Following the application of the liquid rubber, the coated specimens were subjected to curing for 7 days, after which a layer of white latex paint was applied onto the liquid rubber surface by brushing.

### 2.4. Test Protocol

#### 2.4.1. Measurement Locations

**Figure 4:**
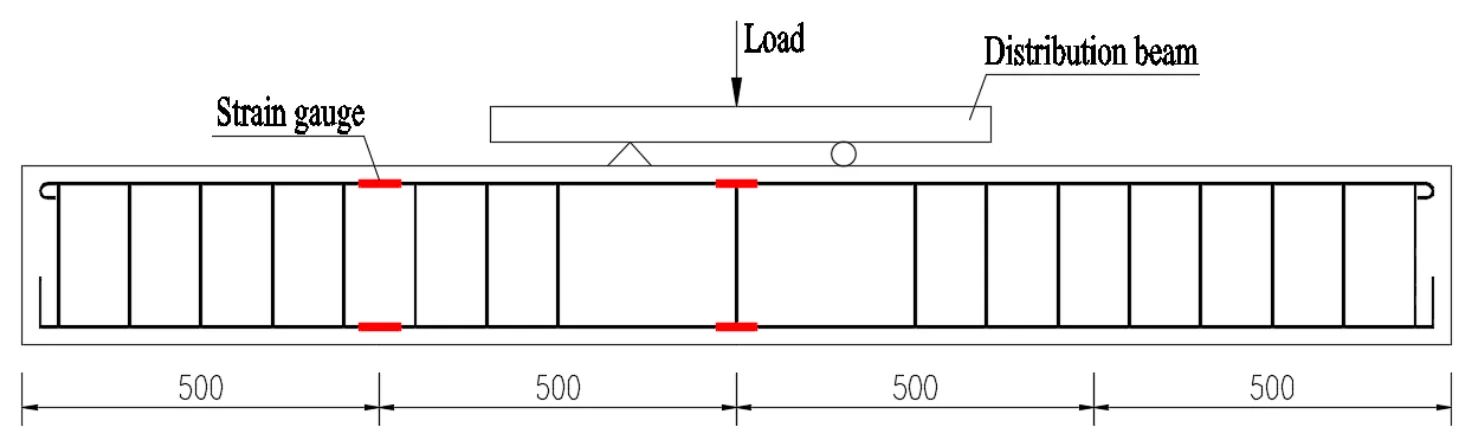
Arrangement of strain gauges on reinforcing bars.

**Figure 5:**
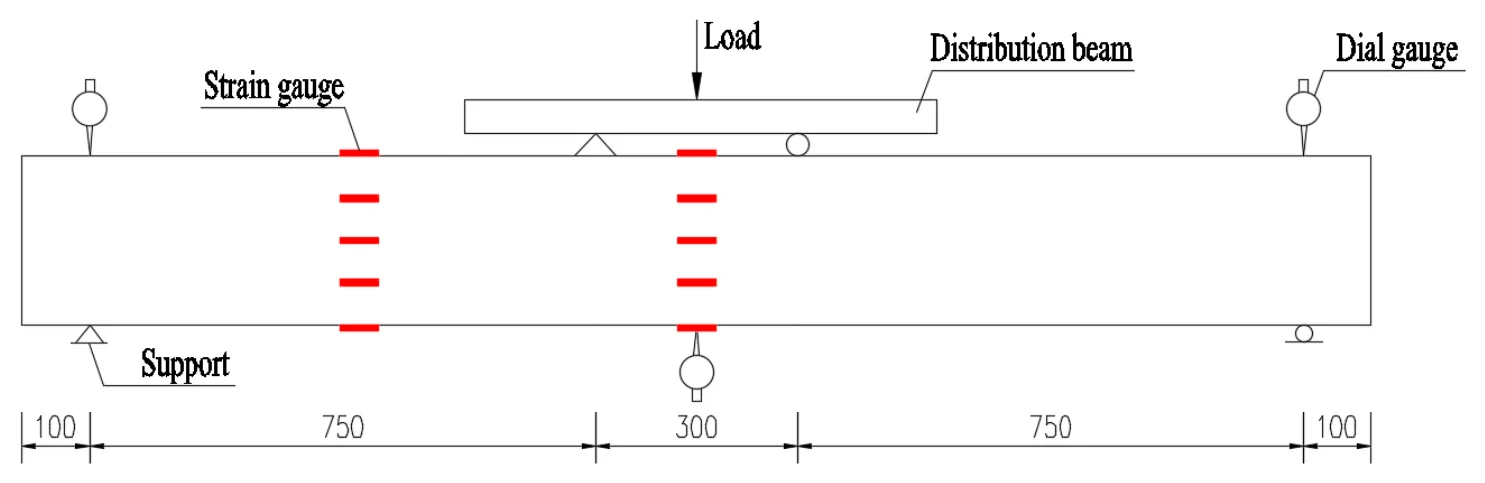
Arrangement of strain gauges on concrete surfaces.

To measure the strain responses of the reinforcement in the pure bending and shear-bending regions, two strain gauges were attached to both the top and bottom longitudinal reinforcing bars at the quarter-span and mid-span sections of each test beam, resulting in a total of eight reinforcement strain gauges. The detailed arrangement of the reinforcement strain gauges is shown in Figure 4. In addition, one dial gauge was installed at the mid-span section and at each support of the test beam to monitor the mid-span deflection and support settlements, respectively. To characterize the strain distribution across the concrete section depth, concrete strain gauges were attached to the top and bottom surfaces at both the quarter-span and mid-span sections of the test beams, with one gauge on each surface. Additionally, three strain gauges were uniformly spaced along the beam height on the side surface at each section, positioned at distances of 62.5 mm, 125 mm, and 187.5 mm from the bottom surface. In total, ten concrete strain gauges were instrumented. Type BX120-5AA (CD Electronic Measuring Technology Co., Ltd., Chengdu, Sichuan, China) strain gauges were used for reinforcing bars, while Type BX120-100AA (CD Electronic Measuring Technology Co., Ltd., Chengdu, Sichuan, China) strain gauges were adopted for concrete surface strain measurements. The specific layout of the concrete strain gauges is illustrated in Figure 5.

#### 2.4.2. Loading Protocol

The test was loaded using a POPWIL MAS-500 hydraulic loading system (Hangzhou Popwil Instrument Co., Ltd., Hangzhou, Zhejiang, China) with a capacity of 50 tons. The concentrated load applied by the actuator was transmitted to the test beam through a load distribution beam, which converted it into two symmetrical concentrated loads. The actuator displacement rate ranged from 0.5 to 50 mm/min, and the load measurement accuracy was within ±0.1 kN. The beam was simply supported, with a shear-span ratio of 3.5, a shear-span length of 750 mm, and a pure-bending span of 300 mm, as illustrated in Figure 5.

Prior to the formal loading, one or two preloading cycles were conducted at a load level not exceeding 50% of the calculated theoretical cracking load. This procedure served to verify the proper operation of all instrumentation, after which the beam was unloaded. The theoretical cracking load of the test beam was calculated according to the Standard for Design of Concrete Structures GB/T 50010-2010 [31], using the formula given in Equation (1). The calculated theoretical cracking moment of the test beam is 4.53 kN·m, corresponding to a theoretical cracking load of 12.08 kN.

***M***_cr_ = ***γf***_tk_***W***_0_ (1)

where ***M***_cr_ is the cracking moment; ***γ*** is the plastic influence coefficient of the section modulus of the concrete member; ***f***_tk_ is the standard value of the axial tensile strength of concrete; and ***W***_0_ is the elastic section modulus of the transformed section at the tensile edge of the member.

**Figure 6:**
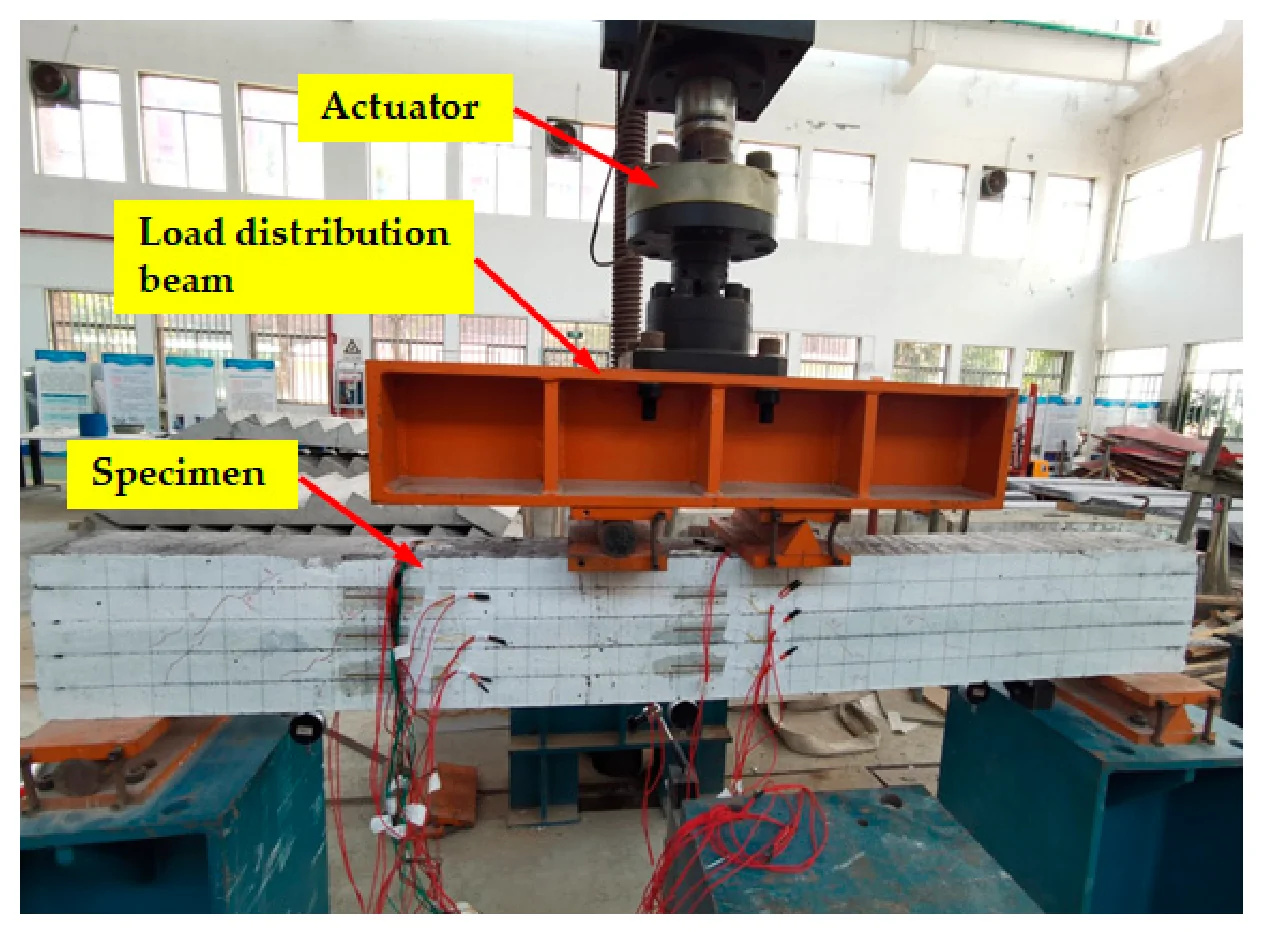
Loading apparatus and the test in progress.

The formal loading was performed under load control with an incremental loading procedure, following the recommendations of GB/T 50152-2012 [32]. A load increment of 5 kN was adopted prior to cracking, which was increased to 10 kN after the onset of cracking, and then reduced to 5 kN after yielding of the longitudinal reinforcement, with loading continued until specimen failure. The tests were conducted at a constant loading rate of 2.5 kN/min. At each load step, the load was maintained for 2–3 min to facilitate the observation of cracks, the recording of dial gauge readings, and the collection of strain data. The loading apparatus and the loading test in progress are shown in Figure 6.

#### 2.4.3. Data Acquisition

The applied load was automatically recorded through the software accompanying the POPWIL loading system (3.2 General Edition). The displacement of the test beam was measured using dial gauges (General Technology Group Harbin Measuring Tools and Cutting Tools Co., Ltd., Harbin, Heilongjiang, China). It should be particularly noted that when the dial gauge needle exhibited continuous fluctuation, the reading was recorded only after it had stabilized. Crack widths were measured with a crack width gauge (Shenzhen Aosvi Optical Instrument Co., Ltd., Shenzhen, Guangdong, China), which had a measurement accuracy of 0.02 mm and a maximum range of 1.6 mm; when the crack width exceeded 1.6 mm, a vernier caliper (General Technology Group Harbin Measuring Tools and Cutting Tools Co., Ltd., Harbin, Heilongjiang, China) was employed instead. Strain data were acquired using a DH3818Y static strain tester (DongHua Testing Technology Co., Ltd., Jingjiang, Jiangsu, China).

## 3. Test Results and Analysis

### 3.1. Failure Mode

**Figure 7:**
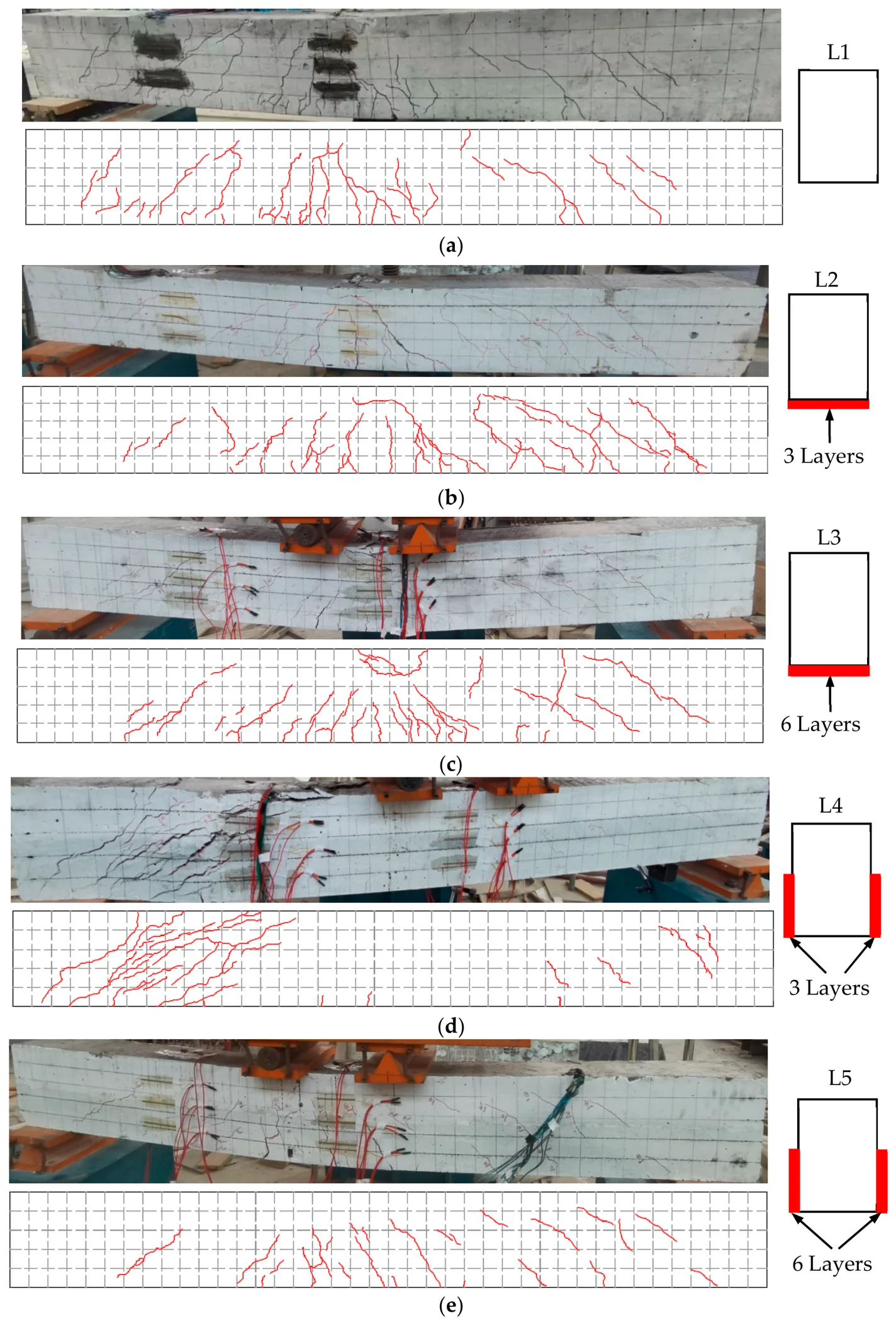

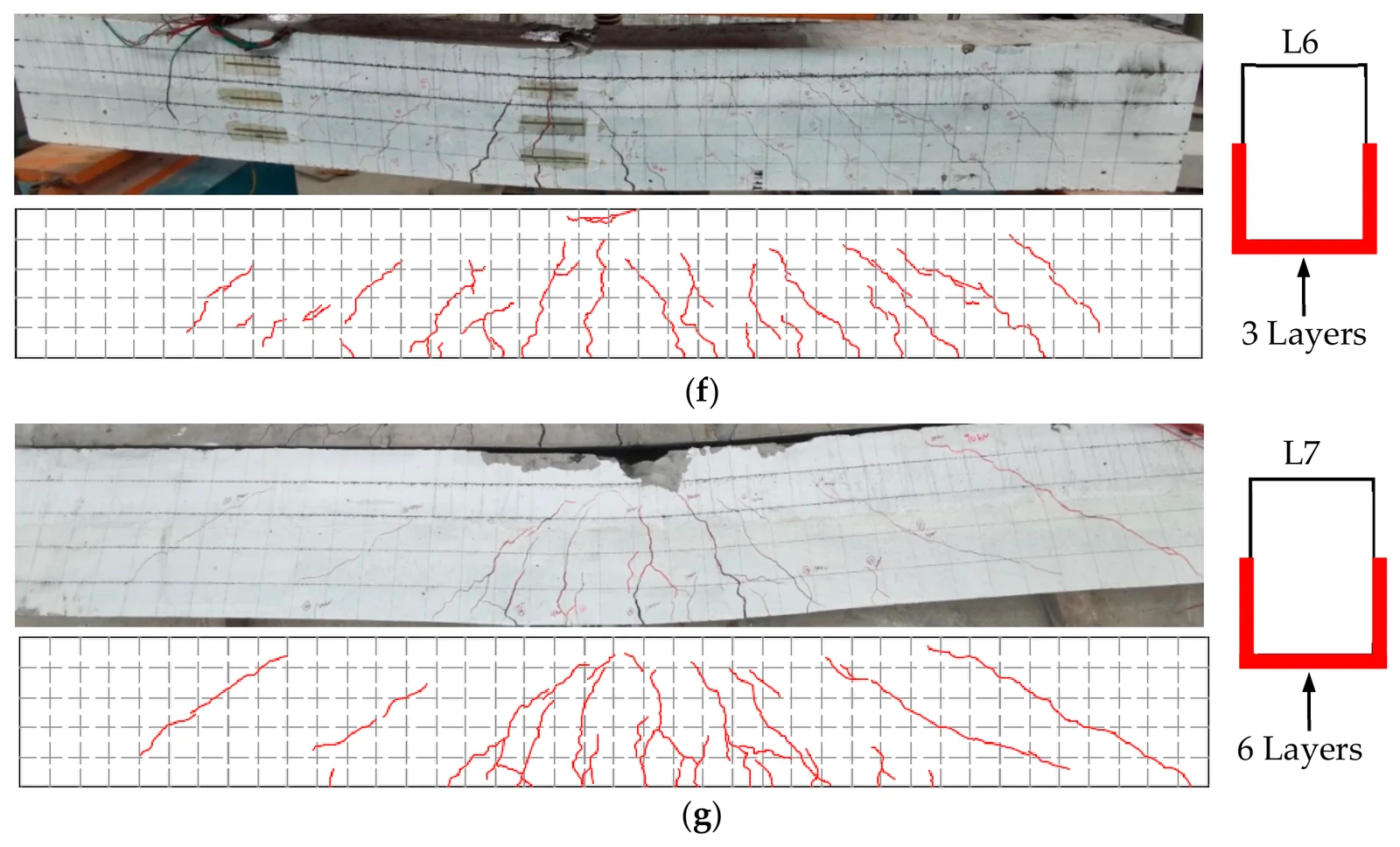
Failure modes and crack patterns of the test beams: (**a**) L1; (**b**) L2; (**c**) L3; (**d**) L4; (**e**) L5; (**f**) L6; (**g**) L7. The red lines in the figure indicate the liquid rubber coating.

The failure modes and crack patterns of the test beams are shown in Figure 7. Specimen L4 failed in shear-compression, whereas the remaining specimens exhibited typical flexural failure, characterized by yielding of the tensile reinforcement followed by crushing of the compressive concrete.

For specimen L4, the first diagonal crack appeared in the shear-bending region at a load of 12.5 kN. As the load further increased, new diagonal cracks continuously developed in this region, and a few vertical cracks also emerged in the bottom tensile zone of the beam. With continued loading, the number of diagonal cracks in the shear-bending region increased progressively, extending obliquely toward the loading points. At a load of 65 kN, the concrete in the shear-bending region was suddenly crushed, leading to the failure of the specimen. Prior to failure, no obvious warning signs were observed, and the longitudinal tensile reinforcement had not yet yielded at the time of failure, with the maximum width of diagonal cracks in the shear-bending region reaching 7 mm. 

Prior to testing, the concrete strength of each specimen was measured using a rebound hammer, and the results are presented in Table 5. As shown in Table 5, the rebound strength of Specimen L4 was 24.9 MPa, which is significantly lower than that of the other specimens. According to the provisions for shear capacity of isolated beams subjected to concentrated loads in GB/T 50010-2010 [31], both the shear-span ratio and concrete strength are critical parameters affecting the shear resistance of reinforced concrete beams. In the present study, the shear-span ratio was 3.5, which is relatively large and consequently has an adverse effect on the shear capacity. Considering the above factors, the shear-compression failure observed in Specimen L4 can be primarily attributed to its lower concrete strength, which led to a reduced shear capacity. When the load reached 65 kN, multiple diagonal cracks had developed in the shear-bending region and extended toward the loading points, substantially reducing the remaining concrete cross-section above the critical diagonal crack. Owing to the relatively low concrete strength, the concrete in the shear-compression zone underwent crushing, resulting in the loss of shear capacity and exhibiting typical characteristics of shear-compression failure. In contrast, the other specimens, with higher concrete strength, maintained the integrity of the shear-compression zone until yielding of the longitudinal tensile reinforcement occurred, thus demonstrating ductile flexural failure.

**Figure 8:**
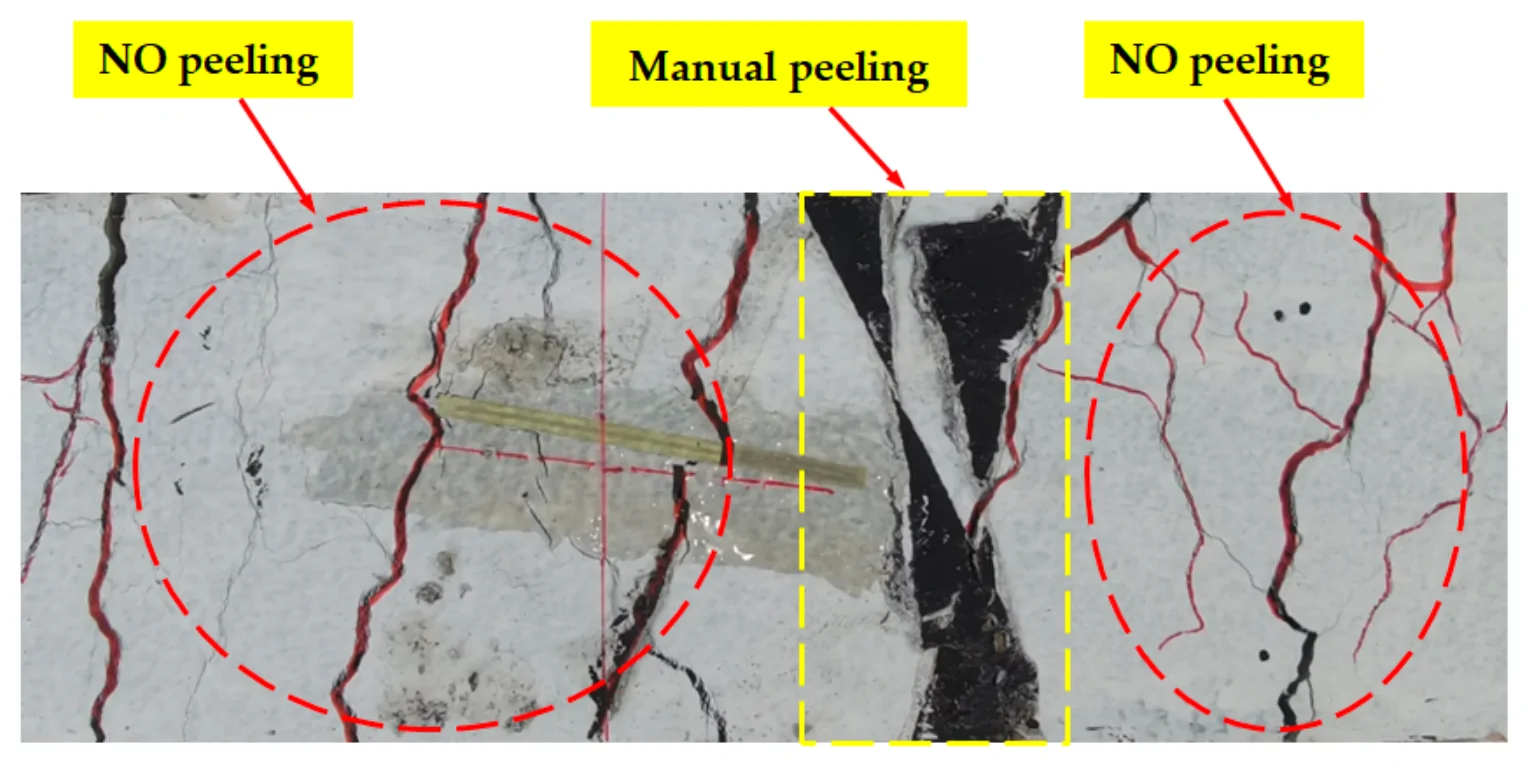
Manual peeling inspection of the liquid rubber coating.

The failure modes of the remaining specimens were generally similar. Taking specimen L3 as a representative example, the first vertical crack appeared at the bottom of the mid-span section at a load of 16 kN. As the load increased, diagonal cracks began to develop in the shear-bending regions, while the vertical cracks at the bottom of the pure bending zone propagated upward. Meanwhile, the diagonal cracks in the shear-bending regions extended obliquely toward the loading points. When the load reached 80.0 kN, the longitudinal tensile reinforcement yielded. With further loading, the cracks at the bottom and on the sides of the beam in the pure bending zone continued to widen. At a load of 87 kN, the concrete at the top of the pure bending zone was crushed, with the maximum crack width on the side face reaching 3 mm. Simultaneously, the deflection of the test beam increased sharply, and the load began to decrease, indicating the occurrence of failure. Prior to failure, this specimen exhibited clear warning signs and demonstrated satisfactory ductility. Post-test manual peel examination of the liquid rubber coating revealed satisfactory interfacial adhesion between the coating and the underlying concrete substrate. No significant delamination or peeling was observed throughout the inspected area, as illustrated in Figure 8.

### 3.2. Load-Bearing Capacity and Ductility

The characteristic loads and corresponding mid-span displacements of the test beams are summarized in Table 6. In the table, *F*_cr_ denotes the cracking load, *F*_y_ represents the load corresponding to the yielding of the longitudinal tensile reinforcement, and *F*_u_ is the ultimate load. Δ_y_ is the mid-span deflection of the test beam at the yielding of the reinforcement, while Δ_u_ is the mid-span deflection at the ultimate load. The ductility coefficient μ is defined as the ratio of Δ_u_ to Δ_y_.

It should be noted that the cracking load defined in this study corresponds to the load at the initial micro-cracking stage of the concrete, rather than the load at which the cracks become visible on the liquid rubber coating surface. When the concrete initially cracks, the crack width is extremely small (typically on the order of 0.05–0.1 mm). Owing to its high elasticity, the liquid rubber coating can fully accommodate this minor cracking through its own tensile deformation; therefore, no obvious crack marks can be observed on the coating surface at this stage. As the load continues to increase, the concrete cracks further develop and the crack width gradually enlarges. When the localized tensile deformation exceeds the ultimate elongation of the liquid rubber, the coating begins to undergo localized tearing at the locations corresponding to the cracks. Only at this point do the cracks gradually become visible on the coating surface.

As shown in Table 6, the application of liquid rubber coating on either the bottom or side surfaces of the beams improved the cracking load to a certain extent. When three layers of liquid rubber were applied, the cracking loads of the coated beams were generally comparable to those of the uncoated specimen L1, showing no significant improvement. However, when six layers were applied, a marked increase in the cracking load was observed for all coated beams. Specifically, compared with specimen L1, the cracking loads of specimens L3, L5, and L7 increased by 28.0%, 35.2%, and 56.8%, respectively. These results suggest a certain correlation between the increase in cracking load and the thickness of the liquid rubber coating. To explore the underlying mechanism, the potential tensile contribution of the coating was estimated based on its tensile strength, thickness, and effective width using the following equation:

***F*** = ***σ***_t_·(***t***·***b***) (2)

where ***F*** is the theoretical maximum tensile force provided by the coating; ***σ***_t_ is the tensile strength of the liquid rubber; ***t*** is the total coating thickness; ***b*** is the effective width of the coating. The estimation indicates that the theoretical maximum tensile forces provided by the three-layer (0.6 mm) and six-layer (1.2 mm) coatings are approximately 0.171 kN and 0.342 kN, respectively. Even if these forces were fully mobilized, they are considerably smaller than the observed increases in cracking load (3.5 kN for L3, 4.4 kN for L5, and 7.1 kN for L7, compared with L1). This clearly demonstrates that the direct tensile resistance of the liquid rubber coating itself is far too limited to account for the observed increase in cracking load. Therefore, the increase in cracking load is more reasonably attributed to the formation of a continuous protective membrane by the liquid rubber coating on the tensile zone of the beam, which enables cooperative deformation with the concrete substrate and joint resistance to tensile stresses. When too few layers are applied, the film is too thin to establish an effective continuous protective layer, thereby contributing little to the cracking load. Once the coating reaches a sufficient thickness (e.g., six layers), it forms an integral continuous membrane on the tensile zone, allowing cooperative load transfer between the liquid rubber and the concrete. Moreover, the liquid rubber exhibited excellent toughness, with an elongation at break of 870%, enabling it to accommodate substantial localized tensile strains without premature rupture during the cooperative deformation process.

The analysis of yield loads of the test beams indicates that the application of liquid rubber coating had little influence on the yield load. This is primarily because, after the initiation of concrete cracking, the tensile stresses in the tension zone were mainly sustained by the longitudinal tensile reinforcement, whereas both the concrete and the liquid rubber layer were no longer able to effectively participate in tensile load sharing. Since all test beams were reinforced with identical longitudinal tensile reinforcement, their yield loads were generally comparable. For Specimen L4, the longitudinal tensile reinforcement had not yet yielded at failure due to the premature occurrence of shear-compression failure.

Overall, the application of the liquid rubber coating on the exterior of concrete beams did not exhibit a clear enhancement effect on the ultimate load. This is primarily attributed to the fact that the ultimate load is governed by the yield strength of the longitudinal tensile reinforcement and the compressive strength of concrete, neither of which can be effectively improved by the external coating. Although specimen L7 exhibited an ultimate load (92.7 kN) that was approximately 6.6% higher than that of specimen L1 (87.0 kN), this difference cannot be statistically validated due to the single-specimen configuration for each test parameter.

**Figure 9:**
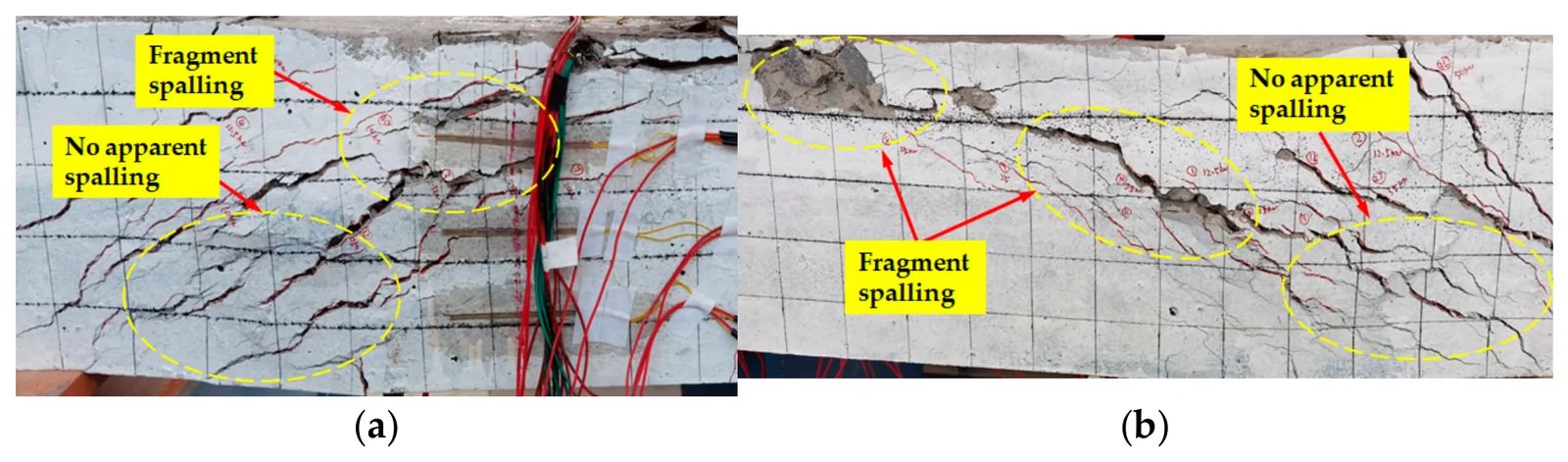
Detailed failure views of specimen L4: (**a**) front side; (**b**) back side.

As shown in Table 6, the ductility coefficients of the test beams coated with liquid rubber were all higher than those of the uncoated specimen. Among them, specimens L5 and L7 exhibited the most significant improvements, with increases of 38.8% and 34.9%, respectively, compared with specimen L1. This indicates that the external application of liquid rubber can enhance the ductility of the test beams to a certain extent, and the degree of improvement is closely related to the number of coating layers—specimens with six layers showed better ductility enhancement than those with three layers. This improvement can be attributed to the following mechanism: after yielding of the longitudinal tensile reinforcement, cracks on the side and bottom surfaces of the beams widened considerably, stretching the liquid rubber coating. During the later loading stages, the rubber membrane on the coated specimens was visibly stretched and remained continuous across the major cracks, creating a “bridging” effect that provided a certain degree of confinement to the spalled concrete fragments and delayed their complete detachment. Taking specimen L4 as an example (Figure 9), the liquid rubber was applied only to the lower half of the beam side surface. In contrast to the upper uncoated region, where numerous concrete fragments were observed to have spalled off, the lower coated region exhibited no significant detachment of fragments, which remained largely confined by the stretched rubber membrane. These observations suggest that, owing to its favorable extensibility, the liquid rubber coating may have contributed, to some extent, to delaying the abrupt load drop caused by concrete crushing, thereby increasing the deformation capacity of the specimens prior to failure, which is reflected in an improvement in ductility.

### 3.3. Concrete Strain

**Figure 10:**
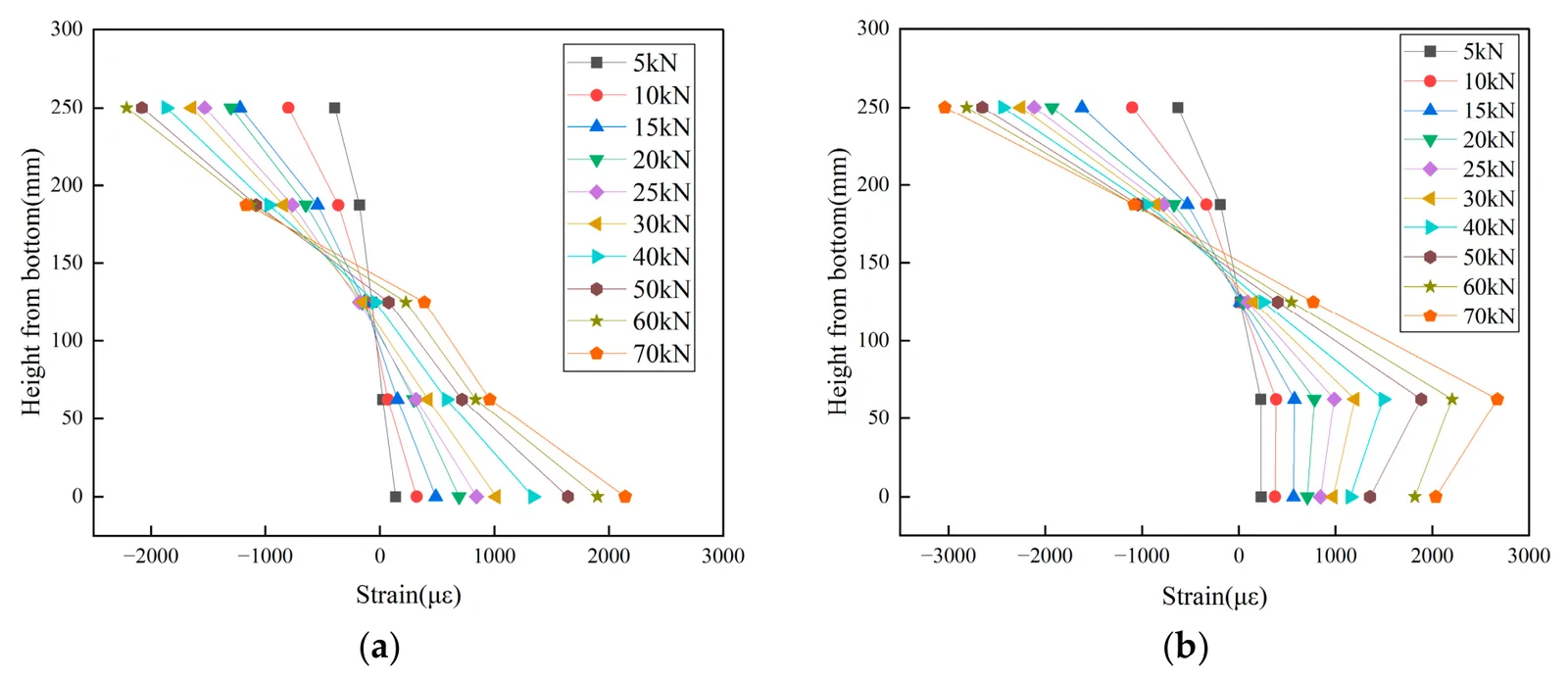

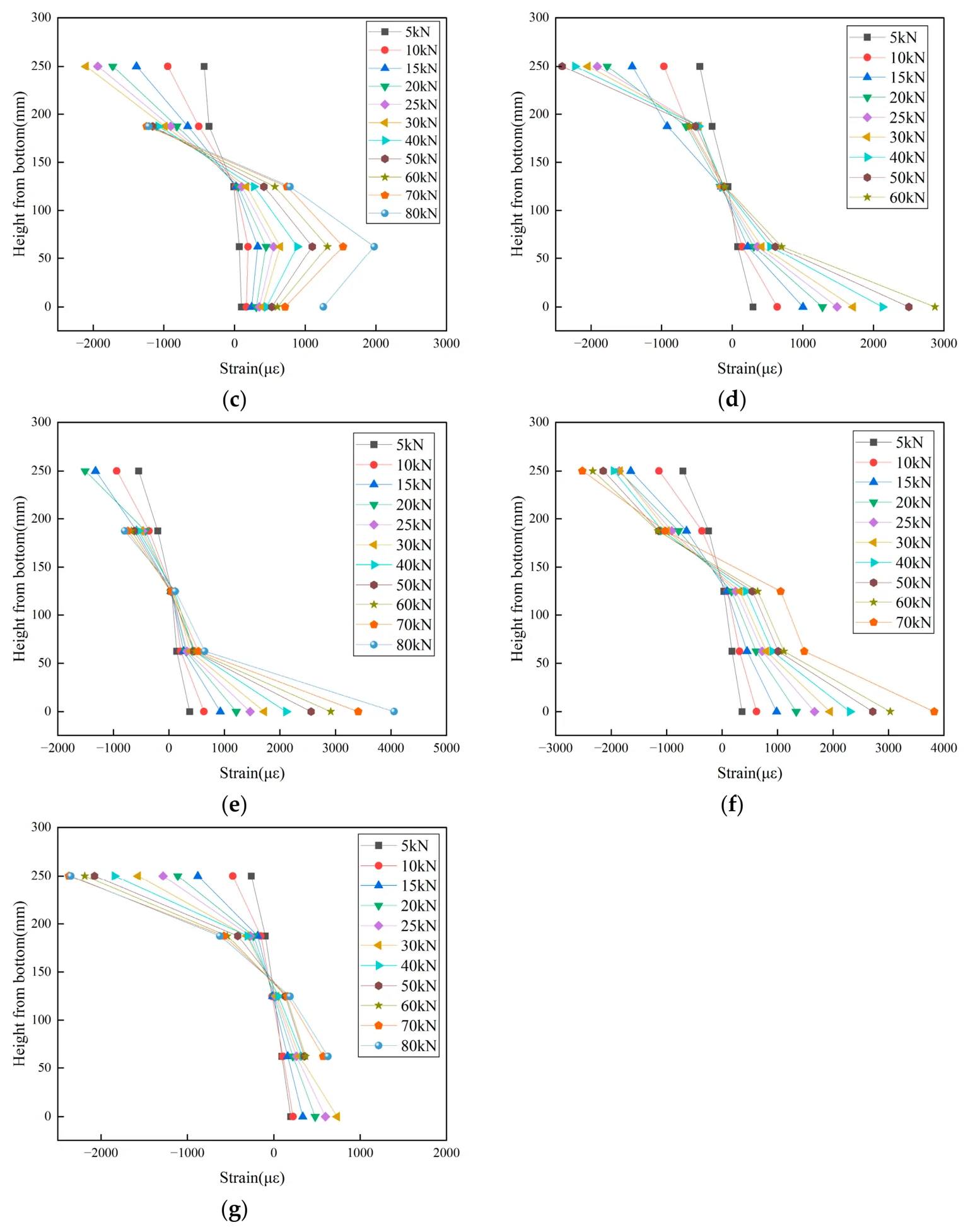
Distribution of concrete strain along the beam depth: (**a**) L1; (**b**) L2; (**c**) L3; (**d**) L4; (**e**) L5; (**f**) L6; (**g**) L7.

Figure 10 presents the distribution of concrete strain along the cross-sectional height at the mid-span of each test beam under various load levels. The missing strain data for some specimens were caused by damage to the corresponding strain gauges during the tests.

As shown in Figure 10, the compressive strains in the concrete of all specimens were approximately linearly distributed along the beam depth. At lower load levels, the neutral axis was located approximately 125 mm from the bottom of the beam. As the load increased, the tensile strain at 125 mm from the beam bottom gradually increased, accompanied by an upward shift of the neutral axis.

With the exception of specimens L2 and L3, the tensile strains in the concrete of the remaining specimens were approximately linearly distributed along the beam depth, generally conforming to the plane-section assumption for the pure bending zone of reinforced concrete beams. However, for specimens L2 and L3, the distribution of tensile strain along the beam depth gradually deviated from linearity with increasing load, and the concrete strain at the beam bottom was smaller than that at 62.5 mm from the bottom. This phenomenon may be primarily attributed to the application of liquid rubber coatings on the bottom surfaces of L2 and L3 (three and six layers, respectively), which enveloped the concrete surface and provided a certain confining effect on the deformation of the bottom concrete, thereby reducing the strain at the beam bottom.

For specimens L4 and L5, the liquid rubber was applied to the lower half of both side surfaces, which provided a certain confining effect on the deformation of the concrete in the tensile zone on the side faces. As a result, the concrete strain at the beam bottom was considerably larger than that on the side faces in the tensile zone. Furthermore, the restraining effect on the side-face tensile concrete was more pronounced in specimen L5 (six layers) than in specimen L4 (three layers), indicating that the confinement effectiveness on concrete deformation improves with increasing thickness of the liquid rubber coating.

It should be acknowledged that the observed nonlinear strain distribution may also be influenced by factors such as cracking, localized strain concentration, strain gauge positioning, or measurement uncertainty. Nevertheless, several lines of evidence suggest that these alternative factors alone are unlikely to fully account for the observed trends. Strain redistribution induced by cracking would be expected to occur in both coated and uncoated specimens, yet the nonlinear behavior was observed exclusively in the bottom-coated specimens (L2 and L3). Localized strain concentration typically leads to elevated readings near crack locations; in contrast, the present results show a reduction in strain at the beam bottom relative to the adjacent gauge, which is more indicative of a confining effect. Moreover, all strain gauges were carefully installed at predetermined positions, and the measurement system was calibrated following standard procedures, rendering systematic errors an unlikely explanation for the consistent differences observed between coated and uncoated specimens.

Based on the strain distribution characteristics discussed above, the presence of the liquid rubber coating may have altered the stress boundary conditions at the surface layer of the concrete beam. Consequently, the conventional plane-section assumption may not be fully applicable to the locally confined tensile zone wrapped by the flexible material. This indirectly suggests that the coating may participate in the cooperative load transfer mechanism and delay the strain development in the tensile concrete to some extent.

### 3.4. Steel Strain

**Figure 11:**
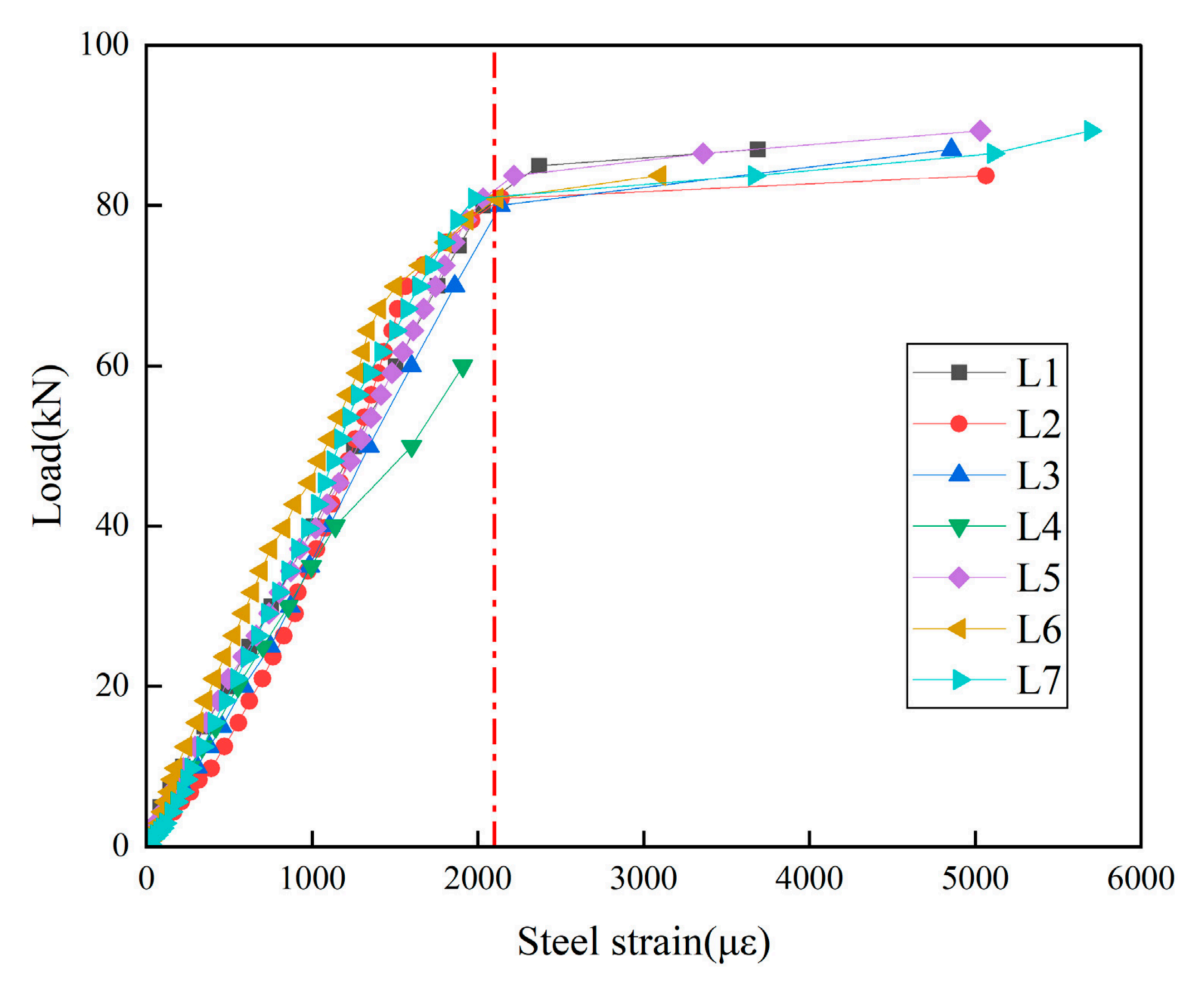
Load–strain curves of longitudinal tensile reinforcement at mid-span of the test beams.

Figure 11 presents the load versus longitudinal tensile reinforcement strain curves at the mid-span of the test beams. As shown in the figure, the longitudinal tensile reinforcement reached yielding in all specimens except L4, which is consistent with the macroscopic failure modes—specimen L4 exhibited shear-compression failure, whereas the remaining specimens displayed typical flexural failure.

Moreover, except for specimen L4, the strain evolution trends of the longitudinal reinforcement were generally similar among the specimens. Before the yield load was attained, the reinforcement strain increased approximately linearly with the applied load, and the slopes of the curves were nearly identical for all specimens. The yield load of each specimen was approximately 80 kN. After yielding, the reinforcement strain increased sharply, accompanied by a distinct yield plateau. 

To evaluate the influence of the liquid rubber coating on the mechanical response of the longitudinal tensile reinforcement, strain values at four representative load levels, namely 20, 40, 60, and 80 kN, were extracted and statistically analyzed, as summarized in Table 7. The coefficient of variation (COV), defined as the ratio of the standard deviation to the mean, was adopted as the primary metric to assess data scatter. At 20 kN, the COV was 16.8%, which is relatively high, mainly attributable to the initial load seating of the testing machine and the stabilization process of the strain gauges at the early stage of loading. These phenomena are commonly observed in flexural tests of reinforced concrete beams. As the load increased, the COV decreased to 9.9% at 40 kN and 12.9% at 60 kN, and further dropped to 3.5% at 80 kN. This decreasing trend in COV with increasing load indicates that the initial scatter was gradually overshadowed as the specimens entered a more stable loading regime. The above results suggest that the load–strain responses of the longitudinal tensile reinforcement were generally consistent across all specimens, implying that the liquid rubber coating had a limited effect on the strain development of the longitudinal tensile reinforcement.

### 3.5. Load–Deflection Curves

**Figure 12:**
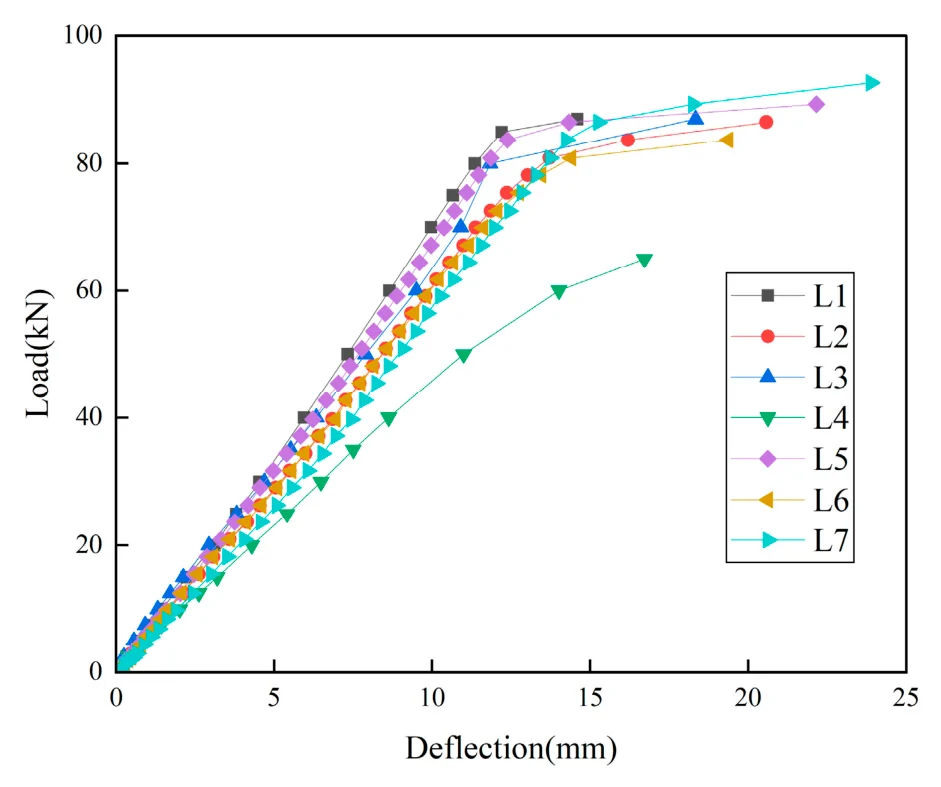
Load–deflection curves at mid-span of the test beams.

Figure 12 presents the load versus mid-span deflection curves of the test beams. Except for Specimen L4, the load–deflection curves of the remaining specimens exhibited generally similar trends and could be divided into three stages. The first stage extended from the onset of loading to concrete cracking, during which the deflection increased linearly. The second stage ranged from concrete cracking to yielding of the longitudinal reinforcement, where new cracks continuously emerged and existing cracks propagated and widened, accompanied by a gradually increasing rate of deflection growth. The third stage spanned from reinforcement yielding to specimen failure, during which the deformation of the longitudinal reinforcement increased sharply after yielding, leading to a rapid rise in beam deflection, eventually followed by crushing of the compressive concrete and subsequent failure.

**Figure 13:**
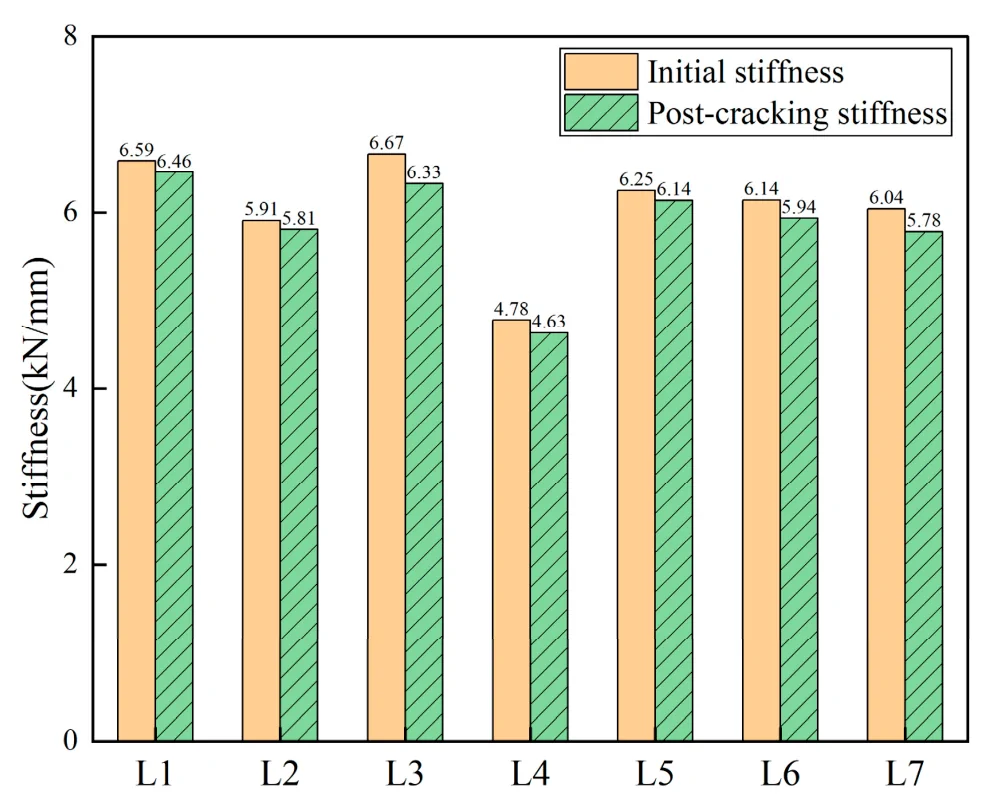
Initial stiffness and post-cracking stiffness of the test beams.

To investigate the stiffness evolution of the test beams at different loading stages, both the initial stiffness and the post-cracking stiffness were evaluated. The initial stiffness ***K***_i_ was taken as the slope of the linear portion of the load–deflection curve from the onset of loading up to concrete cracking. The post-cracking stiffness ***K***_c_ was defined as the secant slope at 0.7 times the ultimate load ***F***_u_, i.e., ***K***_c_ = 0.7***F***_u_/Δ, where Δ denotes the corresponding mid-span deflection. This load level was selected because all specimens had developed substantial cracking while the longitudinal reinforcement had not yet yielded, indicating a stable post-cracking serviceability stage. Comparing the secant stiffness at this consistent load level provides a reasonable measure of the average flexural capacity under serviceability conditions and ensures objectivity and consistency across different specimens. The calculated results of initial and post-cracking stiffness are presented in Figure 13. As shown, the post-cracking stiffness of all specimens is slightly lower than the initial stiffness, which is consistent with the fundamental mechanism that tensile concrete becomes inactive after cracking, leading to a reduced sectional moment of inertia. Except for specimen L4, the difference in initial stiffness between the liquid rubber-coated specimens and the uncoated ones falls within ±10%, which is likely attributable to the inherent variability of the concrete material. Comparative analysis of stiffness reduction before and after cracking reveals that the coated and uncoated specimens exhibit essentially identical stiffness loss. Accordingly, the external application of liquid rubber has a negligible effect on the flexural stiffness of the test beams and cannot effectively enhance it.

### 3.6. Limitations and Future Research

The present study has several limitations that merit further investigation.

(1) Only a single specimen was tested per condition, with no replicates, precluding systematic statistical analysis of data scatter and individual variability. Future research should include replicate specimens to corroborate the observed trends.
(2) Direct measurement of the relative slip between the liquid rubber coating and the concrete substrate was not performed, nor was the restraining effect of the coating on crack propagation quantified. Subsequent studies may employ displacement transducers at the coating–concrete interface or adopt digital image correlation (DIC) techniques to better elucidate the interfacial synergy between the coating and the concrete.
(3) The long-term durability of the liquid rubber coating, including its degradation under wet–dry cycles, freeze–thaw cycles, and carbonation, has yet to be systematically evaluated. Further research should involve long-term exposure tests to assess its performance degradation under service conditions.
(4) The thermal performance and fire resistance of the liquid rubber coating at elevated temperatures or under fire conditions have not been addressed in this study. As a polymeric material, liquid rubber may undergo softening, thermo-oxidative degradation, loss of adhesion, or even thermal decomposition at elevated temperatures, thereby compromising its protective efficacy for concrete structures. Future research should systematically evaluate its thermal stability and fire resistance [33] across a range of temperature conditions.

## 4. Conclusions

This study carried out flexural tests on reinforced concrete beams externally coated with liquid rubber to evaluate its effects on failure mode, characteristic loads, ductility coefficient, sectional strain distribution, and stiffness. The main conclusions are as follows:

(1) All specimens exhibited typical flexural failure, except L4, which failed in shear-compression mode owing to its lower concrete strength. The liquid rubber coating showed satisfactory bond with the concrete substrate; no peeling or debonding was observed throughout loading up to failure.
(2) The external application of liquid rubber exhibited a certain enhancing effect on the cracking load of concrete beams, and the extent of this enhancement was related to the coating thickness. Compared with the uncoated control specimens, the cracking loads of beams coated with six layers on the bottom surface, on the side surfaces, and on both the bottom and side surfaces increased by 28.0%, 35.2%, and 56.8%, respectively.
(3) The application of liquid rubber coating exhibited a certain improving effect on the ductility of the test beams, with a greater magnitude of increase observed under six coating layers than under three layers. Compared with the uncoated specimens, the ductility coefficients of beams coated with six layers on the side surfaces and on both the bottom and side surfaces increased by 38.8% and 34.9%, respectively.
(4) Spraying liquid rubber on either the bottom or side tensile zones of the beam restrained concrete tensile deformation to a certain extent. When the coating was applied only to the bottom surface, the concrete strain at the bottom was lower than that on the side faces within the tensile zone, resulting in a non-linear distribution of tensile strain along the section height in the pure bending region. This suggests that the conventional plane-section assumption may not be fully applicable to locally confined tensile regions wrapped with flexible materials.

## Figures and Tables

**Table 1:** Liquid rubber coating parameters.

Specimen	Coating Applied	Coating Region	Coating Layers	Coating Thickness (mm)
L1	No	—	—	—
L2	Yes	Full bottom surface	3	0.6
L3	Yes	Full bottom surface	6	1.2
L4	Yes	Lower half of side faces	3	0.6
L5	Yes	Lower half of side faces	6	1.2
L6	Yes	Bottom + lower half of side faces	3	0.6
L7	Yes	Bottom + lower half of side faces	6	1.2

**Table 2:** Mix proportions and mechanical properties of concrete.

Cement (kg·m^−3^)	Water (kg·m^−3^)	Fine Aggregate (kg·m^−3^)	Coarse Aggregate (kg·m^−3^)	W/C	*f*_cu_ (MPa)	*E*_c_ (GPa)
372	186	593	1260	0.5	26.9	29.3

**Table 3:** Physical and mechanical properties of the liquid rubber.

Bonding Strength (MPa)	Tensile Strength (MPa)	Elongation (%)	Curing Time (h)	Density (g·cm^−3^)	Heat Resistance (°C)
0.9	1.90	870	1.63	0.981	110

**Table 4:** Mechanical properties of the reinforcing bars.

Steel Grade	Diameter (mm)	Yield Strength (MPa)	Tensile Strength (MPa)	Elastic Modulus (GPa)	Elongation (%)
HPB300	6	342	439	200	9.91
HRB400	10	459	619	190	19.32
HRB400	16	438	613	208	25.54

**Table 5:** Rebound strength of concrete for each specimen.

Specimen	Rebound Strength (MPa)
L1	27.0
L2	27.7
L3	27.7
L4	24.9
L5	26.3
L6	26.0
L7	29.5

**Table 6:** Characteristic loads and corresponding displacements of the test beams.

Specimen	*F*_cr_ (kN)	*F*_y_ (kN)	*F*_u_ (kN)	Δ_y_ (mm)	Δ_u_ (mm)	*μ*
L1	12.5	80.0	87.0	11.35	14.60	1.29
L2	14.0	80.9	86.5	13.72	20.58	1.50
L3	16.0	80.0	87.0	11.84	18.34	1.55
L4 *	12.5	—	65.0	—	16.72	—
L5	16.9	83.7	89.3	12.39	22.17	1.79
L6	12.5	80.9	83.7	14.40	19.37	1.35
L7	19.6	80.9	92.7	13.74	23.88	1.74

* The longitudinal tensile reinforcement of this specimen had not yielded at failure.

**Table 7:** Mid-span longitudinal tensile reinforcement strains under selected load levels.

Specimen	Reinforcement Strain (με)			
	20 kN	40 kN	60 kN	80 kN
L1	493	1009	1503	2032
L2	701	1076	1434	2141
L3	587	1106	1599	2136
L4 *	552	1140	1908	—
L5	494	1022	1549	1999
L6	411	836	1308	2116
L7	544	972	1372	1980
Mean (με)	540.3	1023.0	1524.7	2067.3
COV (%)	16.8	9.9	12.9	3.5

* This specimen failed at 65 kN and was excluded from the analysis at 80 kN.

## Data Availability

The original contributions presented in this study are included in the article. Further inquiries can be directed to the corresponding author.
